# Knowledge and attitude of healthcare prescribers and pharmacists toward antimicrobial stewardship program and the barriers for its implementation

**DOI:** 10.1186/s13756-024-01382-9

**Published:** 2024-04-02

**Authors:** Anan S. Jarab, Tasneem O. AL-Alawneh, Osama Y. Alshogran, Shrouq Abu Heshmeh, Tareq L. Mukattash, Yara A. Naser, Eman Alefishat

**Affiliations:** 1 Department of Clinical Pharmacy, Faculty of Pharmacy, Jordan University of Science and Technology, 22110 Irbid, P.O. Box 3030, Jordan; 2 College of Pharmacy, AL Ain University, Abu Dhabi, United Arab Emirates; 3 School of Pharmacy, Medical Biology Centre, Queen’s University Belfast, Northern Ireland, 97 Lisburn Road, BT9 7BL Belfast, UK; 4 Department of Medical Sciences, College of Medicine and Health Science, Khalifa University of Science and Technology, 127788 Abu Dhabi, United Arab Emirates; 5 Department of Biopharmaceutics and Clinical Pharmacy, Faculty of Pharmacy, The University of Jordan, 11942 Amman, Jordan; 6 Center for Biotechnology, Khalifa University of Science and Technology, 127788 Abu Dhabi, United Arab Emirates

**Keywords:** Antimicrobial stewardship program, Healthcare prescribers, Pharmacists, Knowledge, Attitude, Barriers

## Abstract

**Background:**

Antimicrobial stewardship (ASP) is considered a key prevention strategy in addressing the worldwide concern of accelerating antimicrobial resistance. Limited research is available regarding healthcare providers’ knowledge and attitude toward antimicrobial stewardship and the barriers for its implementation.

**Methods:**

The present cross-sectional study was conducted on pharmacists and healthcare prescribers (HCPs) in different hospital sites across Jordan. A validated survey was used to evaluate HCPs and pharmacists’ knowledge, and attitudes towards ASP and the barriers for its implementation. Logistic and linear regression were conducted to identify the factors associated with knowledge and attitude toward ASP, respectively.

**Results:**

A total of 603 participants, 69 (11.4%) pharmacists and 534 (88.6%) HCPs completed the study questionnaire, with a response rate of 80.4%. The overall mean knowledge about ASP was 7.16 out of 10, ranging from 0 to 10 (SD 2.22). Being a pharmacist and increased awareness/familiarity about ASP were associated with improved ASP knowledge. The overall average attitude score was = 3.8 ± 0.49 (range: 1.8–4.8). Results revealed that being a pharmacist and improved knowledge were associated with improved attitude toward ASP. Lack of specialized staff with expertise in ASP and lack of access to education and training programs were the major barriers hinder ASP implementation.

**Conclusion:**

Despite the reasonable knowledge and the positive attitude toward the ASP, several barriers were reported, particularly by the pharmacists. Therefore, promoting the presence of adequately skilled healthcare personnel, creating easily accessible online courses, and establishing a comprehensive database of ASP resources are all suggested approaches to improve the application of ASP in healthcare settings.

## Introduction

Antimicrobials agents can treat many infectious diseases as they have the ability of not only improving a patient’s quality of life, but have proven to be improve survival in several severe infective conditions [1]. However, the inappropriate use of antimicrobial agents leads to the development of antimicrobial resistance (AMR) [2]. AMR is a major public health problem worldwide, which leads to increase in morbidity, mortality, and medical costs [2]. The World Health Organization predicts 10 million deaths in 2050 if the problem is not being addressed by all stakeholders [3]. The centre for disease prevention and control (CDC) estimated that AMR requires $20 billion direct healthcare costs and $35 billion due to loss of productivity annually [4]. In 2021, the CDC published first-ever estimates demonstrating that the yearly cost of treating infections caused by six multidrug-resistant bacteria in healthcare exceeds $4.6 billion in the United States [5]. 

Antimicrobial Stewardship Program (ASP) is considered a key prevention strategy in addressing the worldwide concern of accelerating AMR [6, 7]. A meta-analysis revealed that the application of hospital-based ASP was associated with several improvements in clinical and financial outcomes, including a decrease in the amount of antibiotics used, the total cost of antibiotics, the number of infections caused by a particular antibiotic-resistant pathogen, and the length of hospital stay [8]. 

The implementation of effective ASP programs requires a well-equipped and knowledgeable multidisciplinary healthcare team consisting of physicians, pharmacists, microbiologists, epidemiologists, and infectious disease specialists with sufficient experience in their respective fields [9]. Physicians play a critical role in shaping antimicrobial policy, crafting prescribing guidelines, and establishing antimicrobial approval structures [10], while pharmacists play an important role in the implementation of ASP via optimizing antibiotic selection and regimens [11]. To ensure their active engagement in such a process, it is imperative to understand their perspectives, knowledge, and attitudes towards ASP.

Limited research is available regarding healthcare providers’ understanding and perspectives regarding ASP in Jordan and worldwide. A previous study concluded that the Jordanian practitioners still need an educational intervention to improve their knowledge regarding ASP [12]. The current study aimed at exploring health care prescribers’ (HCPs) and pharmacists’ knowledge and attitudes toward ASP and the barriers for its implementation. Findings should provide an insight for health authorities to develop strategies and implement interventions for effective implementation of the ASP by the pharmacists and the HCPs at different clinical settings. Additionally, examining the knowledge and attitudes of HCPs regarding ASP can reveal areas where the multidisciplinary team collaboration and communication could be improved.

## Methods

### Study design and subjects

A cross sectional questionnaire-based study was conducted in the period from September through December 2021 using a convenience-sampling technique. The questionnaire was distributed in English via WhatsApp community groups that included only the HCPs and pharmacists in each study site and the survey access was restricted to the email addresses of the participants to mitigate the possibility of duplicate survey access. The HCPs were physicians of different specialities and dentists working at Royal Medical Services (RMS) hospitals (Prince Rashid Hospital and AL Hussein Hospital), King Abdullah University Hospital and Princess Basma Hospital. These hospital settings are classified as tertiary hospitals and located in the north and the middle of Jordan. The hospitals play a crucial role in delivering the majority of healthcare services to residents in these areas. In addition, the hospitals serve as a suitable representation of governmental, military, and educational hospitals in Jordan.

### Study instrument

A custom-designed questionnaire was used to collect socio-demographics of the participants including age, gender, marital status, profession, education level, years of experience and department of work. The participants were also asked if they were aware/familiar with ASP or if they received a previous ASP training..

The 10-item knowledge part was designed after a thorough review of the relevant published studies [13, 14]. This part assessed the HCPs and pharmacists’ knowledge about different aspects of ASP using true, false and don’t know possible answers to each item. In scoring the questionnaire, each correct answer was given a score of one and each incorrect answer was given a score of zero. The answer “don’t know” was considered as incorrect. The scores were then summed for a total score ranging from 0 to 10. For the purpose of the present analysis, the participants were divided into two groups: those scoring more than the mean score were considered having sufficient knowledge about ASP, and those scoring less than the mean score were considered to have insufficient knowledge.

The 15-item attitude domain was adapted from earlier studies with rephrasing of some statements [14, 15]. The participants were asked to respond to this domain items using a five-point Likert scale ranging from strongly disagree to strongly agree. The positive attitude statements (items 1–7) were scored from 5 for strongly agree to 1 for strongly disagree, while the negative attitudes statements (items 8–10) were scored from 5 for strongly disagree to 1 for strongly agree. The mean attitude score was calculated for each statement where higher scores indicating more positive attitude.

The last part, which was adapted from an earlier Saudi study, [15] consisted of eight items evaluating the participants’ barriers to implement ASP. The participants were asked to answer the questions in this domain on a five-point Likert scale ranging from strongly disagree (score 1) to strongly agree (score 5). The survey was reviewed by experts in the field of infectious diseases including infection control physicians and professors in pharmaceutical microbiology and changes including rewording and refining the questionnaire were made where appropriate. Next, the survey was piloted on twenty HCPs and pharmacists to obtain feedback on the appropriateness of the questionnaire with regard to its content, length and suitability. Piloted sample was not included in the final analysis of this study.

### Statistical analysis

Data were analysed and coded using the Statistical Package for the Social Sciences (IBM SPSS) version 25. Continuous variables were described in terms of mean (standard deviations), while categorical variables were described in terms of frequency and percentages. The independent sample t test or Chi-square test was used to investigate the association between different variables and the dichotomous outcome ASP knowledge. Binary logistic regression analysis was performed to identify significant and independent predictors of ASP knowledge. The independent sample t test, ANOVA, chi-square and Pearson correlation were used to find the association between different variables and the outcome attitude score as appropriate. Multiple linear regression was performed to explore factors independently associated with attitude score. Fisher exact test and chi square test were used to measure the difference between pharmacists and HCPs with regard to barriers reporting. Variables with a *P* value less than 0.2 in the univariate analysis were selected for inclusion in the multivariate regression models. A cut off *P* value < 0.05 was used to identify significant predictors of ASP knowledge and attitude in the multivariate models.

## Results

Out of 750 HCPs and pharmacists who were invited to participate in the study, 603 participants, 69 (11.4%) pharmacists and 534 (88.6%) HCPs completed the study questionnaire, with a response rate of 80.4%. The mean age (SD) of the participants was 30.3 (0.24) years. Most of the study participants were males (71.3%), a quarter working in the surgery and urology departments (25.9%), and about half had less than or equal to four years of practice (57.4%). The majority did not receive a previous ASP training (92.5%), and about half were not aware about ASP (55.7%). Others sociodemographic characteristics of the participants are presented in Table 1.

**Table 1:** Sociodemographic characteristics of the study participants (*n* = 603)

Characters	Frequency (%)
Gender	
Male	430 (71.3)
Female	173 (28.7)
Marital status	
Married	284 (47.1)
Others (single, widowed, etc.)	319 (52.9)
Profession	
Healthcare prescribers (Physicians and dentists)	534 (88.6)
Pharmacist	69 (11.4)
Years of experience	
< 4 years	346 (57.4)
5–9 years	167 (27.7)
More than 10 years	90 (14.7)
Hospital department	
Internal medicine	123 (20.4)
General surgery and urology	156 (25.9)
Dental medicine	53 (8.8)
Special surgery	86 (14.3)
Pharmacy	48 (8.0)
Emergency and radiology	50 (8.3)
Genecology and paediatrics	60 (10.0)
Others ^a^	27 (4.5)
Work place	
Prince Rashid Military Hospital	266 (44.1)
King Abdullah University Hospital	156 (25.9)
Basma Hospital	77 (12.8)
Royal medical services (AL Hussein) Hospital	104 (17.2)

^a^ Others: Internship, operating room, Psychiatry, observation, pathology, aviation medicine and neurosurgery

The mean knowledge score was 7.16 out of 10, ranging from 0 to 10 (SD 2.22). As shown in Table 2, the participants demonstrated sufficient knowledge about some aspects of ASP, particularly de-escalation therapy to avoid resistance (84.1%), the importance of the availability of experts in antimicrobial therapy (87.1%) and the necessity to receive regular feedback and evaluation from the experts on regular basis (83.1%). Other participants reported insufficient knowledge with other aspects, particularly knowledge about the role of ASP in reducing potential side effects (56.4%), and antimicrobial cycling (47.8%).

**Table 2:** Knowledge about ASP (*n* = 603)

Item ^a^	Correctly Answered N (%)
1- The antimicrobial stewardship program (ASP) helps to enhance antimicrobial prescribing by the appropriate selection of antimicrobial regimen.	433 (71.8)
2-ASP helps reducing antimicrobial resistance.	458 (76)
3-ASP helps reducing antimicrobial side effects.	340 (56.4)
4- The Defined Daily Dose (DDD) is the average of maintenance dose per day for a drug used for its main indication in adults.	404 (67)
5- Antimicrobial cycling employs antimicrobial rotation of a particular drug with scheduled substitutions that exhibits a comparable spectrum of activity.	288 (47.8)
6- Transition from broad-spectrum antimicrobial drug to narrow spectrum is important to reduce to antimicrobial resistance problem.	507 (84.1)
7-Time-sensitive automatic stop orders strategy reduces the antimicrobial resistance problem.	383 (63.5)
8- Feedback and reviews by an expert in antimicrobial use have been highly effective in optimizing antimicrobial therapy.	501 (83.1)
9-Prescription of some antimicrobial drugs require the availability of expertise in antibiotics use and infectious disease.	525 (87.1)
10- According to the ASP, the patient can stop the medication before completing the full course if symptoms improve.	477 (79.1)

^a^ The correct answer for questions 1–9 is (True), question 10 is (False)

Results of the univariate analysis showed that being a pharmacist, increased years of experience, awareness about ASP, and having previous training on ASP were significantly associated with sufficient knowledge about ASP (*P* < 0.05). The practice site was not associated with knowledge of ASP (*P* = 0.728). As shown in Table 3, results of regression analysis showed that the odds of having sufficient ASP knowledge among pharmacists were 2.46 times the odds among the HCPs. Furthermore, the odds of having sufficient ASP knowledge among participants who had previous awareness about ASP were 2.72 the odds in those who were unaware about ASP.

**Table 3:** Multivariate logistic analysis of the factors associated with knowledge about ASP

Variable	Beta	Odds ratio	CI	*P* value#
Age	-0.011	0.99	0.94–1.04	0.645
Gender				
Female	Ref	Ref	Ref	Ref
Male	0.124	1.13	0.74–1.74	0.574
Profession				
Healthcare prescribers (Physicians and dentists)	Ref	Ref	Ref	Ref
Pharmacists	0.9	2.46	1.28–4.75	0.007
Years of experience				
< 4 years	Ref	Ref	Ref	Ref
5–9 years	-0.19	0.83	0.54–1.27	0.385
> 10 years	0.381	1.46	0.65–3.3	0.359
Familiarity with ASP	1.001	2.72	1.9–3.89	< 0.001
Previous ASP training	0.378	1.46	0.66–3.21	0.348

#The significant *P* value was set as < 0.05

The overall mean attitude score was 3.8 ± 0.49, ranging from 1.8 to 4.8. As shown in Table 4, the participants demonstrated positive attitude toward different aspects of ASP. In particular, they believed that ASP is necessary to reduce AMR (86.9%) and to improve prescription practice (86.1%). They also believed that effective implementation of ASP requires the engagement of physicians and pharmacists who are expert in ASP (84.3%), and could provide clear instructions about antibiotic use (84.4%), particularly with regard to restriction of antimicrobial use (83.5%). Although most of participants believed that antimicrobial resistance could be minimized (80.3%) and they were willing to implement and improve ASP practice in their hospital setting (81.9%), the majority considered AMR as a challenging practice (55.9%) and they were not confident enough to prescribe antimicrobials according to ASP regulations (33.1%).

**Table 4:** Attitudes towards ASP (*n* = 603)

Attitude/ perception	Strongly disagree (N %)	Disagree (N %)	Neutral (N %)	Agree (N %)	Strongly agree (N %)	Mean score
1- The control of antimicrobial resistance is a global necessity.	47 (7.8)	7 (1.2)	25 (4.1)	143 (23.7)	381 (63.2)	4.3 ± 1.1
2- Improving antimicrobial prescribing should be an organizational priority.	29 (4.8)	11 (1.8)	44 (7.3)	272 (45.1)	247 (41.0)	4.2 ± 0.98
3- Healthcare professionals who deal with antimicrobial by ordering, dispensing, administration, and monitoring should be involved in ASP education program.	21 (3.5)	15 (2.5)	127 (21.1)	279 (46.3)	161 (26.7)	3.9 ± 0.94
4- There’s a need to have physicians and pharmacists who are specialist in prescribing the appropriate antimicrobial agent.	30 [5]	7 (1.2)	58 (9.6)	255 (42.3)	253 (42.0)	4.2 ± 0.99
5-There’s a need to have an approval process for the prescribing of selected antimicrobials to certain clinical condition in a hospital.	25 (4.1)	15 (2.5)	59 (9.8)	306 (50.7)	198 (32.8)	4.1 ± 0.95
6-Providing detailed instructions on proper use of antibiotics could help in minimizing antimicrobial resistance.	24 [4]	16 (2.7)	54 [9]	298 (49.4)	211 (35.0)	4.1 ± 0.95
7- I would be willing to participate in any activities to improve the quality of antimicrobial use at my hospital.	12 [2]	7 (1.2)	90 (14.9)	330 (54.7)	164 (27.2)	4.0 ± 0.8
8- Antimicrobial resistance cannot be controlled or minimized. ^a^	174 (28.9)	310 (51.4)	57 (9.5)	49 (8.1)	13 (2.1)	4.0 ± 0.95
9- Antimicrobial resistance represents a challenge for me in my daily practice. ^a^	16 (2.7)	42 [7]	208 (34.5)	247 (41)	90 (14.9)	2.4 ± 0.92
10- I don’t feel confident in my ability to prescribe antibiotics without increasing antimicrobial resistance. ^a^	33 (5.5)	161 (26.7)	209 (34.7)	169 [28]	31 (5.1)	3.0 ± 0.99

^a^ Negative attitude statements were reversely scored

Results of the univariate analysis showed that increased knowledge score, female gender, being a pharmacist, working in KAUH and the RMS hospital, increased years of experience, being unaware about ASP and not having previous training on ASP were significantly associated with improved attitude toward ASP (*P* < 0.05). As shown in Table 5, results of the multiple linear regression revealed that being a pharmacist increased the attitude score by 0.226 as compared with other ASP practitioners. Furthermore, each unit increase in the knowledge score was associated with 0.009 increase in the attitude score. Working at KAUH or the RMS hospital increased the attitude score by 0.095 and 0.115 respectively. On the other hand, having previous awareness about ASP and receiving training about ASP were negatively associated with ASP attitude score.

**Table 5:** Multivariate analysis of the factors associated with the attitude toward ASP

Variable	Beta	SE	CI	*P* Value#
Knowledge score	0.029	0.009	0.047–0.112	0.001
Gender				
Female	Ref	Ref	Ref	Ref
Male	-0.034	0.048	-0.129–0.609	0.483
Profession				
Healthcare prescribers	Ref	Ref	Ref	Ref
Pharmacists	0.226	0.07	0.088–0.364	0.001
Hospital				
RMS (Prince Rashid Hospital)	Ref	Ref	Ref	Ref
King Abdullah University Hospital	0.095	0.047	0.002–0.187	0.046
Basma Hospital	-0.109	0.06	-0.228–0.009	0.07
RMS (AL Hussein Hospital)	0.115	0.054	0.009–0.222	0.034
Unawareness of ASP	0.149	0.042	0.067–0.231	< 0.001
No previous ASP training	0.243	0.082	0.082–0.403	0.003

#The significant *P* value was set as < 0.05

The most commonly reported barriers for ASP implementation were lack of specialized staff (70%) and lack of education programs about ASP (67.4%). Except for lack of ASP understanding by the hospital administration and lack of financial support, all the other barriers were significantly more reported by the pharmacists when compared with other HCPs.

## Discussion

The current study participants reported reasonably sufficient knowledge and they were willing to implement ASP in clinical setting. Nevertheless, the participants, particularly the pharmacists, reported several barriers for ASP implementation. When compared with a recent study conducted in Jordan and evaluated physicians and pharmacists’ attitudes and practice of ASP, [12] the present study should provide a deeper understanding and a broader insights about the information needs, and how the pharmacists and prescribers understand and perceive ASP in addition to the barriers they experience for ASP implementation.

Most of the current study participants were not conversant with ASP and did not receive a training on ASP and its implementation. A study conducted in a Ghanaian Tertiary Hospital reported that pharmacists, medical doctors, nurses and medical laboratory scientists who received a training on ASP showed an improved knowledge about ASP and AMR, which was demonstrated by reduced empirical antibiotic prescribing [7]. 

Although the participants showed sufficient knowledge about some aspects of ASP, some of the participants reported insufficient knowledge with regard to the role of ASP in reducing potential side effects, the defined daily dose (DDD), time-sensitive automatic stop orders strategy and antimicrobial cycling. An earlier study showed that restricting antibiotics use, which is recommended by the ASP, help controlling potential antibiotics side effect and AMR [14]. A study conducted in China showed that implementing DDD and time stop order strategy results in reduce antibiotic consumption [16]. Antibiotic cycling is a practice whereby multiple antibiotic classes are used in various hospital environments to minimize the emergence of resistance that might occur because of using a single or a limited number of antibiotic classes [17]. Several studies reported antibiotic cycling as one of the most effective strategies for reducing AMR and the potential side effects. A study conducted in France showed that the strategy of antibiotic rotation reduced the incidence of late-onset hospital-acquired pneumonia, reduced mortality, and reduced antibiotic resistance [18]. Other studies reported minimization of ventilator-associated pneumonia after antibiotic cycling among ICU patients [19, 20]. 

Results showed that being a pharmacist and increased awareness about ASP were significantly associated with improved ASP knowledge. Pharmacists are well versed in the pharmacology of antibiotics and they are expected to review prescriptions and advise physicians on the optimum selection of antibiotics [21]. The positive association between awareness and knowledge about ASP in the present study was also reported in earlier studies [6, 7, 14, 22]. 

Although the current study participants showed good attitude toward different aspects of ASP, some participants perceived antimicrobial resistance as a challenge in their daily practice and others were not confident in prescribing antibiotics without increasing antimicrobial resistance. An earlier study showed that the majority of the physicians and pharmacists believed that AMR is a major challenge in the clinical practice [23]. Another study showed that some of the participating HCPs were not confident about their knowledge and practice in the area of antimicrobial prescribing [15]. Although the majority of the current study participants believed that AMR can be controlled or minimized, higher percentages were reported in earlier studies [24]. The identified negative attitudes towards ASP implementation, as shown by participants’ responses to attitudes statements in Table 4, may be partially attributed to a lower number of years of clinical experience (less than 4 years) among some individuals.

Being a pharmacist and having sufficient knowledge about ASP were associated with improved attitude toward ASP. Consistent with the current study findings, an earlier study showed that the community pharmacists were more interested in the implementation of collaborative ASP strategies when compared with other health practitioners [25]. Also, KAUH and RMS hospitals are of the largest tertiary hospitals in the region that regularly provide training on different aspects of health services and programs including the ASP, which may justify the increased willingness of the pharmacists and other HCPs who work in these sites to implement ASP in the practice setting. The unexpected observation was the inverse relationship between awareness, training in ASP, and attitude scores. One possible explanation is that pharmacists and HCPs lacking awareness and training on ASP may exhibit greater enthusiasm in implementing the program, seeing it as a potential solution to the prevalent antimicrobial resistance challenges encountered in clinical practice. Conversely, individuals with ASP awareness or specialized training might encounter various obstacles during program implementation, leading to unmet expectations from ASP.

Despite the reasonably good attitude toward ASP, several barriers for ASP implementation were reported in the present study. The majority of the pharmacists and HCPs believed that the lack of ASP specialist staff, lack of access to education and training programs and lack of specialized ASP information resources were the most common barrier for ASP implementation, which agrees with previous findings [15, 26]. The specialist staff is needed to effectively develop the ASP and put appropriate polices for the management of infectious diseases [15]. Lack of knowledge and lower confidence of health care providers to provide ASP, lack of formulary management and funding, and lack of understanding of ASP by hospital administrators were also reported as barriers for ASP implementation in this study, which supports previous research findings [15, 17, 22, 27–31]. When compared with the HCPs, pharmacists in the current study significantly reported more barriers for ASP implementation. An earlier study showed that pharmacists reported high recognition of the importance of the ASP and its impact on reducing the use of antimicrobial agents and the cost of treatments [32]. 

### Strengths and limitations

The current study was conducted on a large sample of participants, including both pharmacists and HCPs, which enhances conclusions that are more robust. In addition, utilizing a validated survey instrument enhances the validity and reliability of the data gathered, thereby boosting the accuracy of the results. Moreover, the knowledge gained from this study provides important direction for upcoming antimicrobial resistance prevention initiatives, and helps in developing effective strategies to minimize AMR and maximize the application of ASP in clinical settings. On the other hand, the current study did not cover all the Jordanian provenances that might limit the generalizability of the findings. The scope of the study was confined to HCPs and pharmacists. It is recommended that future studies broaden their scope to include additional health professions such as nurses who play an integral role in infection prevention and control, and providing collaborative healthcare services. Lastly, the self-reported questionnaire used for data collection could have overestimated responses due to misunderstanding bias.

## Conclusions

The current study showed a margin for improvement in HCPs and pharmacists’ knowledge and attitudes toward the ASP. Healthcare authorities and hospitals administrators should ensure the availability of well-equipped healthcare staff by providing regular educational and training programs, which aim at fulfilling HCPs and pharmacists’ knowledge gaps and improve attitudes toward ASP. The authorities should also focus on overcoming the barriers for ASP implementation in order to enhance the implementation of effective ASP in different healthcare settings in Jordan. This includes the development of accessible online courses, providing individuals with the flexibility to learn at their own. In addition, promoting cooperation between healthcare institutions and professional organizations is advised to establish and preserve a comprehensive database of ASP resources. These efforts should carry the potential to enhance ASP application in the healthcare system and reduce AMR.

## Data Availability

Data generated and/or analysed in the present study are available from the corresponding author on reasonable request.

## Abbreviations

ASP: Antimicrobial stewardship
AMR: Antimicrobial resistance
DDD: Defined daily dose
HCP: Healthcare prescriber
IBM: International Business Machines Corporation
ICU: Intensive-care unit
SPSS: Statistical Package for the Social Sciences
SD: Standard deviation
